# PC, a Novel Oral Insecticidal Toxin from *Bacillus bombysepticus* Involved in Host Lethality via APN and BtR-175

**DOI:** 10.1038/srep11101

**Published:** 2015-06-09

**Authors:** Ping Lin, Tingcai Cheng, Shengkai Jin, Yuqian Wu, Bohua Fu, Renwen Long, Ping Zhao, Qingyou Xia

**Affiliations:** 1 State Key Laboratory of Silkworm Genome Biology, Southwest University, Chongqing, China

## Abstract

Insect pests have developed resistance to chemical insecticides, insecticidal toxins as bioinsecticides or genetic protection built into crops. Consequently, novel, orally active insecticidal toxins would be valuable biological alternatives for pest control. Here, we identified a novel insecticidal toxin, parasporal crystal toxin (PC), from *Bacillus bombysepticus* (*Bb*). PC shows oral pathogenic activity and lethality towards silkworms and Cry1Ac-resistant *Helicoverpa armigera* strains. In *vitro* assays, PC after activated by trypsin binds to BmAPN4 and BtR-175 by interacting with CR7 and CR12 fragments. Additionally, trypsin-activated PC demonstrates cytotoxicity against Sf9 cells expressing BmAPN4, revealing that BmAPN4 serves as a functional receptor that participates in *Bb* and PC pathogenicity. *In vivo* assay, knocking out BtR-175 increased the resistance of silkworms to PC. These data suggest that PC is the first protein with insecticidal activity identified in *Bb* that is capable of causing silkworm death via receptor interactions, representing an important advance in our understanding of the toxicity of *Bb* and the contributions of interactions between microbial pathogens and insects to disease pathology. Furthermore, the potency of PC as an insecticidal protein makes it a good candidate for inclusion in integrated agricultural pest management systems.

During the past decade, interest in isolating bacterial toxins with the hope of identifying novel properties that are particularly suited for the control of agronomically important pests has increased. These toxins have the potential to reduce the use of insecticide sprays, to promote pest suppression, and to involve the incorporation of transgenes that encode these proteins in plants^1^. Many emerging and re-emerging bacterial pathogens have been screened to identify such toxins^2^,^3^. The pore-forming toxins (PFTs) are the largest family of bacterial toxins. According to the type of structure used to interact with the lipid bilayer, PFTs are classified into α-PFT and β-PFT. Colicins^4^ or *Bacillus thuringiensis* (*Bt*) Cry toxins^5^ are representative members of the α-PFT family. *Clostridium perfringens* enterotoxin^6^ and Vcc from *Vibrio cholerae*^7^ are members of the β-PFT family. Additionally, many bacterial toxins that do not fully qualify as PFT are known as AB toxins; in these toxins, the B subunit is responsible for binding to the target cell and for translocating the A subunit into the cytoplasm^8^.

The modes of action of bacterial toxins include damaging cell membranes^2^, inhibiting protein synthesis^9^, activating second messenger pathways^10^, and activating the host immune response^11^. Generally, PFTs bind to a specific receptor, diffuse towards target cell membranes, form multimers, and undergo a conformational change leading to the formation of a pore in the target cell membrane that damages the extracellular matrix^8^. This damage not only results in the loss of membrane function and direct cell lysis but also facilitates bacterial dissemination throughout tissues.

Most bacterial toxins, such as biomphalysin, possess haemolytic function but not oral activity^2^. Therefore, these bacterial toxins are unlikely to be useful insecticides. However, OAIP-1 isolated from venom of the Australian tarantula is highly biologically stable, which likely contributes to its oral activity; therefore, this toxin might have potential as a “standalone” bioinsecticide^12^. Notably, Cry toxins isolated from the bacterium *Bt* have been shown to be orally lethal to some insects. Therefore, Cry toxins have had a widespread and revolutionary impact on insecticide use and crop production, resulting in reduced chemical insecticide use and improved yields^13^,^14^. Nevertheless, the remarkable ability of insects to adapt to insecticides suggests that the evolution of resistance threatens the continued success of *Bt* Cry toxin^15^,^16^. Thus, the development of novel insecticidal toxins that could function as valuable biological alternatives in pest control measures to improve resistance management is of significant interest.

Many species of bacteria that belong to the genus *Bacillus* produce toxins and establish a systemic infection in a variety of hosts^17^. Among them, the pathogen *Bacillus bombysepticus* (*Bb*), which was first identified from sick silkworm larvae cadavers by Hartman^18^, leads to silkworm bacterial black chest septicaemia. However, the pathogenesis of *Bb* is not well understood^19^. Through 16S rRNA gene sequence analysis, *Bb* has been shown to be closely related to *Bacillus cereus* and to *Bt*^20^. Similar to *Bt*, *Bb* also produces spores and parasporal crystal toxin (PC)^20^. Bacterial toxins are often important virulence factors for pathogens^21^. However, no information is available regarding whether PC has pathogenic properties.

*Bb* toxins are thought to poison the silkworm via a mechanism similar to that of the *Bt* Cry toxin, as shown by a genome-wide survey of the host during *Bb* infection^20^. *Bb*-mediated poisoning occurs in the midgut. After oral infection, the PC produced by *Bb* can be digested by gut proteases to generate a toxin fragment. The digested PC can pass through the peritrophic membrane (PM) to bind to the receptors of midgut epithelia cells and damage them. *Bb* can spread to the haemolymph from the damaged midgut. However, no information is available regarding the role of receptors for PC during the pathogenesis of *Bb*.

The interaction between the silkworm, a model lepidopteran organism, and *Bb*, a *Bacillus* organism, represents an important host-pathogen dynamic that could provide insights into the relationship between lepidopteran insects and microorganisms. Here, we isolated an orally active toxin, PC from *Bb*, which plays an important role in insecticidal action. Exposure to PC induces silkworm and *Helicoverpa armigera* death. Our results indicate that interactions with the midgut receptors APN and BtR-175 are key contributors to PC pathogenicity and may be targets for the engineering of an antibacterial silkworm that would reduce economic losses in sericulture production. We propose a schematic model for the process by which *Bb* damages the silkworm midgut and haemolymph, causing death. Specifically, PC binds to midgut receptors, which disrupts homeostasis and material exchange throughout the insect body. Our work, along with studies on *Bt* Cry and other bacteria toxins, suggests that toxin production is a common strategy of entomopathogenic bacteria that interferes with insect guts through interactions with receptors. However, the oral potency of *Bb* PC and its insecticidal effects on *Bt*-resistant *H. armigera* makes it a good candidate for deployment with *Bt* Cry toxins against lepidopterans or other pests in integrated pest management systems that provide levels of crop protection that are more durable.

## Results

### PC is pathogenic to silkworms

**Figure 1:**
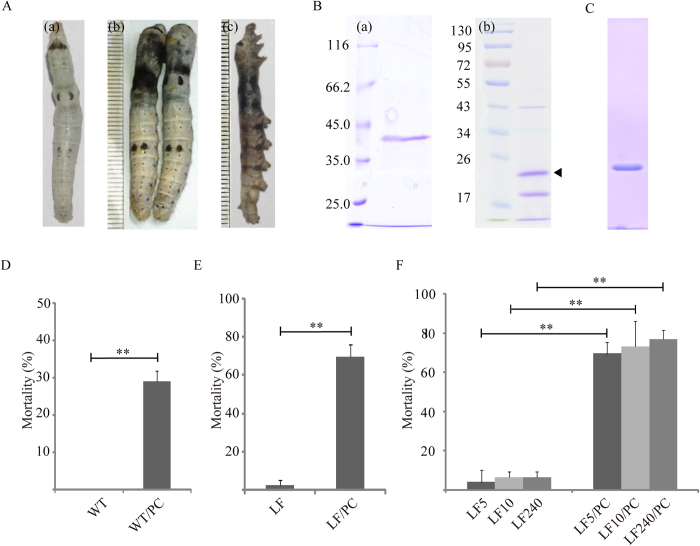
*Bb* parasporal crystal toxin (PC) identified from *Bb* bacterium is pathogenic and causes virulence. (**A**) *Bb* oral infection causes silkworm cuticle peutz and death. The phenotypes of wild-type (**a**), *Bb* treatments at 30 h (**b**), and *Bb* treatments at 40 h (**c**). (**B**) SDS-PAGE analysis of purified PC from *Bb* (**a**) and trypsin-activated PC protein (**b**). PC was extracted from *Bb* cultures according to the purification method used for *Bt* toxin and was digested by trypsin at 37 °C for 6 h. The filled triangle band represents PC after trypsin digestion. (**C**) Purification of PC from a prokaryotic expression system. (**D**) Mortality of newly exuviated 5^th^-instar silkworm larvae following oral infection with PC. WT, unchallenged. (**E**) Mortality observed feeding PC into *Bt*-susceptible *Helicoverpa armigera* (LF) strains. (**F**) The mortality rate of three Cry1Ac-resistant *Helicoverpa armigera* strains characterized to show different resistance levels after oral infection with PC. Error bars depict ±SEM. Statistically significant differences from control samples are indicated; ^**^P < 0.01.

Silkworm larvae were orally infected with *Bb* to induce larval death, leading to black chest septicaemia: a peutz appears on the thoracoabdominal region or the 1^st^ to 3^rd^ segment of the abdomen (Fig. 1Ab) and then expands to encompass the entire body (Fig. 1Ac). To determine whether or not the pathogenic mechanism of *Bb* was associated with PC, PC was purified from bacterial cultures using methods similar to those used to isolate *Bt* parasporal crystals (Fig. 1Ba). Because the protoxin can be hydrolyzed by midgut proteases to form an activated molecule that causes lethal toxicity to insect larvae^22^, the purified PC was digested with trypsin at 37 °C for 6 h and then separated by SDS-PAGE (Fig. 1Bb). All of the bands were excised from the gel and subjected to matrix-assisted laser desorption/ionization-time of flight (MALDI-TOF) mass spectrometry analysis. The filled triangle band represented PC after trypsin digestion, and MALDI-TOF analysis identified only one protein component in this band, namely, a recombinant PC protein that was expressed and purified (Fig. 1C). To confirm the pathogenicity of PC protein, mortality was determined by oral infection of the 5^th^-instar silkworms with 50 μg PC protein per larva. Beginning at 15 h after treatment, the PC-treated larvae began to die (Fig. S1B), but the wild-type (WT) larvae survived (Fig. S1A). We found that PC induced approximately 29% mortality compared to 0% in WT within 4 days (Fig. 1D), with an LD_50_ of 140.512 μg per larva for oral administration at the 5^th^-instar stage. Furthermore, the mortality rate of 3^rd^-instar larvae was 40.3% after 4 days at a dose of 50 μg PC protein per larva. The approximate LD_50_ value calculated for 3^rd^-instar silkworms was 77.35 μg per larva. These results indicated that PC is pathogenic to silkworms.

### Identification of PC pathogenicity towards *Bt*-resistant *H. armigera*

*Bt* crops represent one of the most widespread and controversial applications of pest management^23^. Many studies have addressed pest resistance to *Bt* crops and resistant pests that can overcome the lethality of *Bt* protein^24^. To investigate the pathogenic capacity of PC towards *Bt*-resistant insects for future pest control applications, we performed bioassays on larvae from one *Bt*-susceptible (LF) and three Cry1Ac-resistant *H. armigera* strains (LF5, LF10, and L240). Cry1Ac-resistant *H. armigera* strains were established by Prof. Kongming Wu *et al*. by screening field-collected populations with different doses of Cry1Ac toxins (LF5: 5 μg Cry1Ac/mL artificial diet, LF10: 10 μg Cry1Ac/mL artificial diet, and LF240: 240 μg Cry1Ac/mL artificial diet) under laboratory conditions^25^. PC was fed to 1^st^-instar larvae at a dose of 50 μg toxin/mL artificial diet. PC produced a mortality rate above 60% in the LF species within 5 days (Fig. 1E). Interestingly, three Cry1Ac-resistant strains showed pathogenicities similar to the LF species (Fig. 1F). The mortality rate analysis after oral infection with resistant larvae showed that ~70% of the LF5, LF10, and LF240 infected with PC succumbed within 5 days. The insecticide activity of PC in Cry1Ac-resistant strains indicated its potential use as a bioinsecticide or transgenic crop gene to target *Bt*-resistant insects.

### Molecular characterization of PC

The first 29 amino acids of PC corresponded to a putative signal peptide as predicted by the SignalP program. An analysis of putative post-translational modifications using the NetNglyc and YinOYang servers suggested the absence of N-linked glycosylation and a putativeO-(beta)-GlcNAc glycosylation event at G_74_. To further explore the relationship between PC and *Bt* Cry toxins (Cry1A–Cry28A), we used ClustalX and Mega 4 to visualize phylogenetic relationships. Sequence alignment showed that some sites of PC are conserved with *Bt* Cry toxins (Fig. S2), showing an identity of 8.6% with *Bt* Cry6Aa. However, as to *Bt* Cry6Aa, *Bt* Cry15Aa and *Bt* Cry23A, phylogenetic tree showed that PC resides on its own deep branch (Fig. S3), indicating that it should be considered a distinct toxin. The deduced amino acid sequence of PC displayed limited sequence similarity (≤11.4%) with a range of bacterial pore-forming toxins as determined by a BlastP search.

### Binding assays to detect PC interaction with BmAPN4

*Bt* Cry toxins kill many insects by interacting with receptors such as APN or cadherin, leading to pore formation and ultimately cell lysis^14^,^22^,^26^. *Bb* toxins induce larvae poisoning responses in the midgut that are similar to those induced by *Bt*^20^. Surprisingly, PC accumulation in host intestinal epithelial cells and pores could be observed in the optical membrane of the larvae midgut after *Bb* bacterium infection^20^. A gene encoding an APN protein (BmAPNN4) was obtained by biopanning against *Bb* bacterium from a silkworm midgut T7 phage display cDNA library^27^. Additionally, some midgut-expressed APNs can be modulated by *Bb* infection^20^,^28^. Therefore, we hypothesized that silkworm APNs interact with PC to cause pathogenicity.

**Figure 2:**
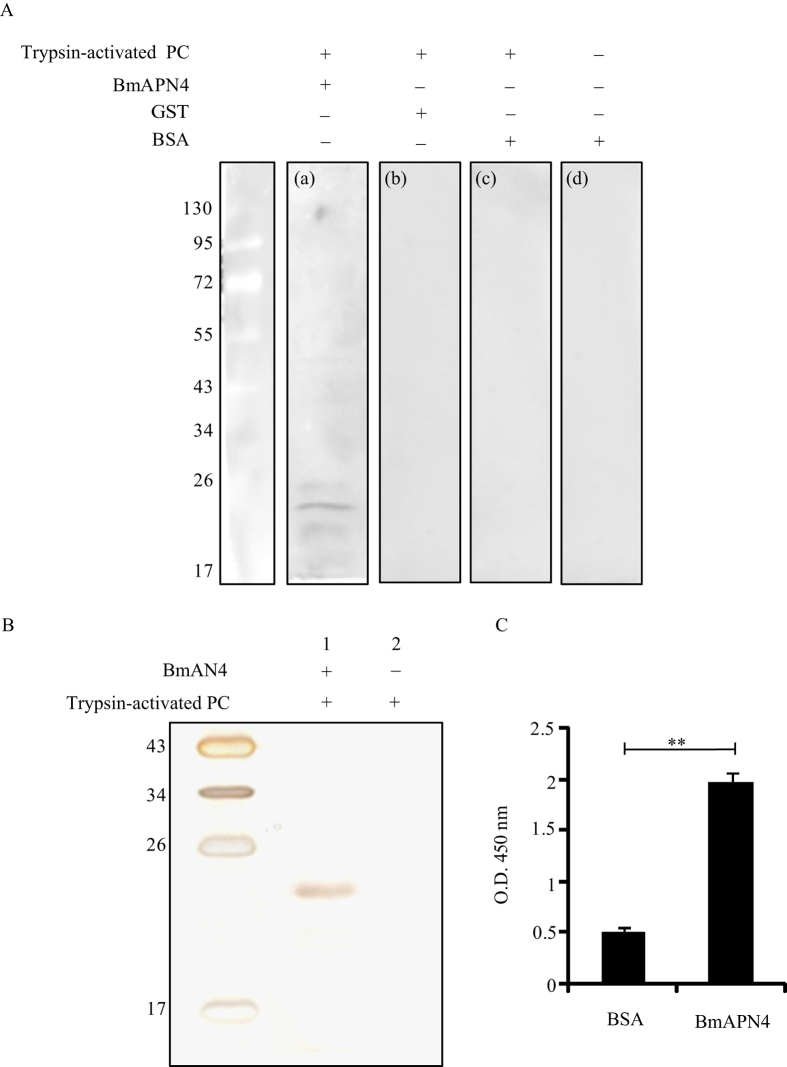
Binding assays for trypsin-activated PC with BmAPN4. (**A**) Far-western blot analysis of trypsin-activated PC and BmAPN4. The trypsin-activated PC was separated by 12% (w/v) SDS–PAGE and transferred to PVDF membranes for far-western blot analysis, in which the recombinant BmAPN4 (**a**), GST (**b**), and BSA (**c**) proteins were incubated with the membranes before adding the anti-GST antibody. The membrane was used for direct immunoblotting using anti-GST antibody (**d**); positive bands were observed only when trypsin-activated PC/BmAPN4 complexes were present. (**B**) A Co-IP assay for trypsin-activated PC and BmAPN4. Lane 1 shows trypsin-activated PC incubated with BmAPN4 that was immunoprecipitated with antibody. Lane 2 shows trypsin-activated PC incubated with antibody as control; the positive band indicates trypsin-activated PC. (**C**) ELISA binding saturation assays of trypsin-activated PC and BmAPN4. Error bars depict ±SEM. Statistically significant differences from the control samples are indicated; ^**^P < 0.01.

To test our hypothesis, far-western blot analysis was performed. The gene encoding BmAPN4 was cloned by RT-PCR, and recombinant BmAPN4 protein with a glutathione S-transferase (GST) tag at the N-terminus was expressed and purified. The trypsin-activated PC proteins were separated and transferred to PVDF membranes. The membranes were separately incubated with BmAPN4 protein before the addition of anti-GST antibody (Fig. 2Aa). Membranes incubated with GST or bovine serum albumin (BSA) protein were used as negative controls (Fig. 2Ab,c), and another negative control, a membrane without PC, was incubated with BSA to exclude the possibility of a direct interaction between the anti-GST antibody and the PVDF membrane (Fig. 2Ad). Positive bands were observed only when trypsin-activated PC/BmAPN4 complexes were present (Fig. 2Aa). Far-western blot analysis with trypsin-activated PC and BmAPN4 showed that trypsin-activated PC was capable of interacting with silkworm BmAPN4. We used co-immunoprecipitation (Co-IP) assays to determine whether BmAPN4 interacted with trypsin-activated PC. As shown in Fig. 2B, the Co-IP assay indicated that PC bound to BmAPN4. Furthermore, ELISA binding saturation assays showed that PC bound to BmAPN4 (Fig. 2C), which confirmed the interaction between PC and BmAPN4.

### Cytotoxic activity of PC in Sf9 cells expressing BmAPN4

**Figure 3:**
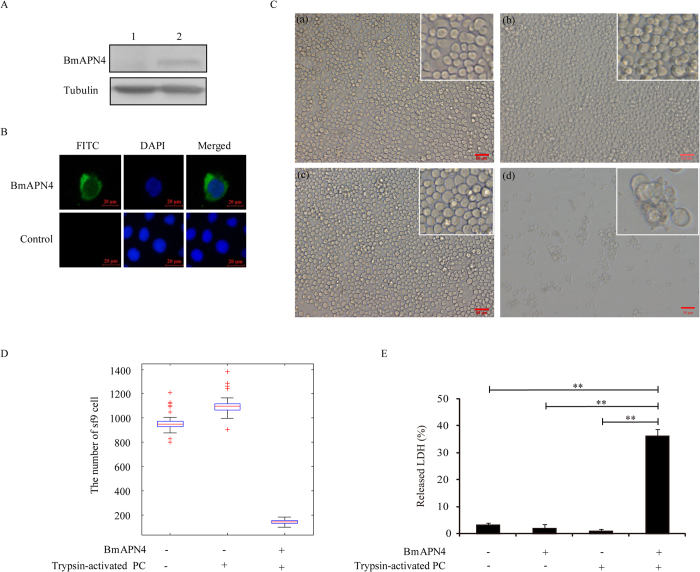
Cytotoxic activity of trypsin-activated PC-induced BmAPN4-expressing Sf9 cells death. (**A**) Western blot analysis of a Sf9 cell line expressing BmAPN4. Membrane proteins were extracted from Sf9 cells after 48 h post-transfection with BmAPN4 expression vector. Lane 1 shows native Sf9 cells as a control. Lane 2 shows BmAPN4 was expressed by Sf9 cells; tubulin was used as an internal control. (**B**) Immunocytochemical analysis of BmAPN4 in Sf9 cells. Signals for Sf9 cells and BmAPN4 were detected under blue and red fluorescence. (**C**) The cytotoxic activity of trypsin-activated PC proteins on Sf9 cells transfected with the gene encoding the BmAPN4 receptor from *Bombyx mori*. Photomicrographs of healthy uninfected cells (**a**), BmAPN4-expressing Sf9 cells (**b**), healthy uninfected Sf9 cells treated with trypsin-activated PC (**c**), and BmAPN4-expressing Sf9 cells treated with trypsin-activated PC (**d**) are shown. Cells that expressed BmAPN4 that were incubated with trypsin-activated PC showed swelling and lysis. (**D**) The number of cells with such alterations after trypsin-activated PC-treated healthy and BmAPN4-expressing Sf9 cells is shown. (**E**) trypsin-activated PC-induced cell death was measured based on the extracellular release of LDH activity after incubation for 6 h. Error bars depict ±SEM. Statistically significant differences from the control samples are indicated; ^**^P < 0.01.

Having determined that PC binds to silkworm BmAPN4, we investigated the biological significance of silkworm BmAPN4 in silkworms infected with *Bb*. To ascertain whether silkworm APNs are involved in PC pathogenicity, the cytotoxic activity of PC in Sf9 cells transfected with BmAPN4 gene was tested. BmAPN4 was expressed in the membrane fractions of Sf9 cells transfected with psl1180[hr3-BmAct4-BmAPN4-SV40] vectors (Fig. 3A, lane 2). Additionally, immunofluorescence was used to evaluate the location of BmAPN4 in cells (Fig. 3B). Separately, the incubation of live, viable cells expressing BmAPN4 with trypsin-activated PC at 50 μg/mL induced distinct morphological changes. A significant proportion of the cells exhibited dramatic cytological changes, including swelling and lysis (Fig. 3Cd). Such effects were not observed in the control Sf9 cells (Fig. 3Ca), Sf9 cells transfected with silkworm BmAPN4 that were not exposed to toxin (Fig. 3Cb), or control Sf9 cells incubated with trypsin-activated PC (Fig. 3Cc), suggesting that the expressed BmAPN4 directly interacted with trypsin-activated PC to induce morphological aberrations and cell lysis. The number of cells with such alterations after PC administration were counted in 50 different optical fields, indicating that the proportion of viable cells was reduced to ~15.8% (Fig. 3D). Susceptibility to trypsin-activated PC was further confirmed using a cytotoxicity assay based on the extracellular detection of LDH activity. Trypsin-activated PC caused a 35.3% increase in the release of total intracellular LDH in BmAPN4-transfected Sf9 cells into the culture medium (Fig. 3E). Untransfected Sf9 cells were not affected by PC. Together with the results of the binding assay (Fig. 2), this finding indicates that BmAPN4 primarily serves as a functional receptor involved in PC pathogenicity.

### Interaction of PC with Bt-R175

**Figure 4:**
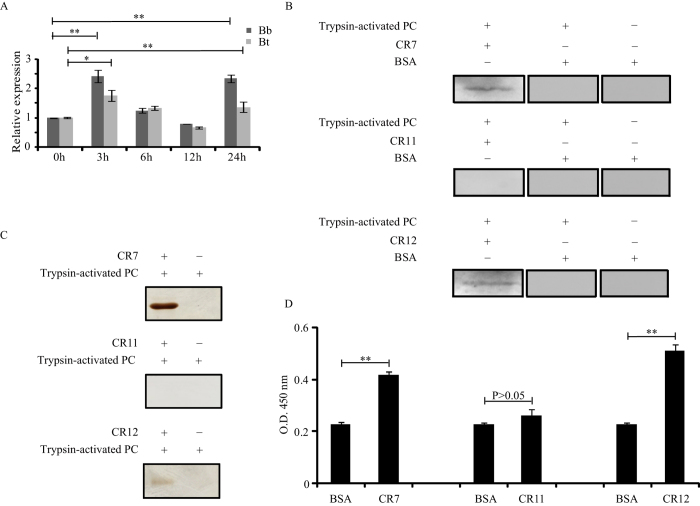
Interaction analysis of trypsin-activated PC with BtR-175 cadherin fragments. (**A**) The induced expression profiles of *BtR-175* after *Bb* and *Bt* challenge by qRT-PCR. (**B**) Far-western blot analysis of trypsin-activated PC and BtR-175 cadherin fragments. The trypsin-activated PC was separated using 12% (w/v) SDS–PAGE and transferred to PVDF membranes for far-western blot analysis, in which recombinant His-CR7, His-CR11, and His-CR12 proteins (lane 1) were incubated with membranes. The membrane was either incubated with BSA (lane 2) or directly immunoblotted (lane 3) as a negative control. Positive bands were observed only when trypsin-activated PC/CR7 and trypsin-activated PC/CR12 complexes were present. (**C**) His-tag pull-down assays for trypsin-activated PC and BtR-175 cadherin fragments. Lane 1 shows BtR-175 cadherin fragments incubated with PC that were digested with trypsin. The bands indicate the pull-down proteins of PC that bound to PC (CR7 or CR12) complexes. Lane 2 shows an agarose gel incubated with trypsin-activated PC as a control. (**D**) ELISA binding saturation assays of trypsin-activated PC and BtR-175 cadherin fragments. Error bars depict ±SEM. Statistically significant differences from the control samples are indicated; ^**^P < 0.01; ^*^P < 0.05.

The interaction with APN concentrates *Bt* Cry toxin in the midgut brush border membrane vesicles (BBMV), where the toxin is able to bind with high affinity to cadherin^29^. Binding to cadherin is complex and involves three cadherin fragments that correspond to the extracellular regions CR7, CR11, and CR12^30^. Therefore, to determine whether cadherin is involved in *Bb* infection in silkworms, qRT-PCR was performed to analyse the expression pattern of cadherin (BtR-175) in the midgut after *Bb* infection. Similar to *Bt* infection, the induced peaks of the gene encoding BtR-175 appeared at 3 and 24 h, corresponding to the induced expression profiles of the gene encoding BmAPN4 after *Bb* infection (Fig. 4A) and suggesting that BtR-175 expression is associated with responses in *Bb*-infected silkworms. However, whether BtR-175 is involved in PC pathogenicity is unknown.

To examine the role of BtR-175 in the process of toxin pathogenicity, we cloned three cadherin fragments that correspond to CR7 (residues M^811^–A^927^), CR11 (residues L^1257^–T^1398^), and CR12 (residues N^1368^–G^1484^) of silkworm BtR-175. The recombinant CR7, CR11, and CR12 proteins with His tags at N-termini were purified for use in binding assays. Far-western blot analysis of trypsin-activated PC and His-CR7, His-CR11, or His-CR12 resulted in positive bands that reacted with anti-His antibody only when trypsin-activated PC/His-CR7 or trypsin-activated PC/His-CR12 complexes were present, indicating that PC bound to CR7 and CR12 cadherin fragments, but not to CR11 (Fig. 4B). The purified His-CR7, His-CR11, and His-CR12 proteins were each incubated with trypsin-activated PC. After incubation, His-CR7-bound protein(s), His-CR11-bound protein(s), and His-CR12-bound protein(s) were purified by affinity chromatography. As a negative control, Ni-NTA beads incubated with trypsin-activated PC were used to exclude the possibility of PC directly binding to the NTA resin. As shown in Fig. 4C, PC was not detected by His-CR11 pull down but could be observed in the CR7 and CR12 pull-down assays. Furthermore, ELISA binding assays confirmed that trypsin-activated PC could bind to CR7 or CR12, but not to CR11 (Fig. 4D). These results showed that PC interacted with silkworm BtR-175 by binding to the cadherin fragments CR7 and CR12.

### Effect of BtR-175 on PC pathogenicity *in B. mori*

**Figure 5:**
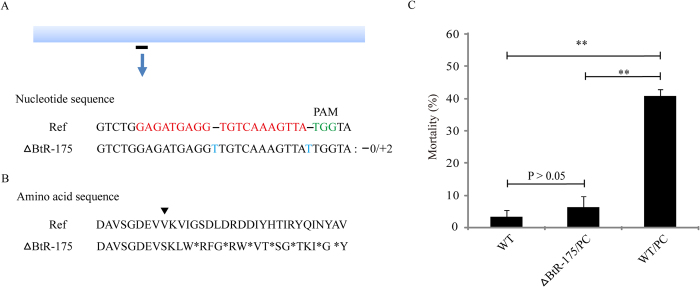
Effect of BtR-175 knock-out in silkworm on PC pathogenicity. (**A**) Cas9/gRNA-induced mutations at the *BtR-175* locus in silkworm. Schematic representation of *BtR-175* and gRNA target sequences (sequences at the bottom). The wild type sequence (Ref) is shown. gRNA sites are highlighted in red and PAM sequences are shown in green. Sequences of mutations at the targeted *BtR-175* locus in G_3_ silkworm (ΔBtR-175) and 2 bp sequence was inserted into the target site by TA-clone sequencing. (**B**) Amino acid sequences of mutations. ^*^: terminator. (**C**) The mortality of 3^rd^-instar ΔBtR-175 larvae after oral infection with PC. Error bars depict ±SEM. Statistically significant differences from the control samples are indicated; ^**^P < 0.01.

To further characterize BtR-175 biological function during the toxin pathogenicity, we used CRISPR/Cas9 genome editing to knock out the *BtR-175* gene for bioassay. A guiding sequence of *BtR-175* gRNA was synthesized and inserted into pUC57-gRNA to form pUC57-BtR-175. The plasmids pUC57-hA4-Cas9 and pUC57-BtR-175 were introduced into silkworm embryos by co-microinjection to induce BtR-175 mutagenesis. Finally, homozygous mutants were obtained from G3 moths using T7EI genotyping and TA clone sequencing to reveal the exact mutant sequences (Fig. 5A). A homozygous mutant (ΔBtR-175) was inserted into 2 bp with disrupted *BtR-175* leading to translation termination and loss-of-function (Fig. 5B). In total, 50 μg PC per larva was used to calculate the mortality of 3^rd^-instar ΔBtR-175 strain for oral administration toxicity assays. Remarkably, ΔBtR-175 silkworms were much more potent against PC. The mortality of WT silkworms orally infected with toxins at 4 days (40.2 ± 2.4%) was significantly elevated (^**^P < 0.01) compared to that of the ΔBtR-175 strain (5.9 ± 3.25%) (Fig. 5C). Moreover, no significant mortality was observed in WT silkworms (2.8 ± 2.4%) and in the ΔBtR-175 strain. This result indicated that knocking out *BtR-175* gene increased the resistance of silkworms to PC. Together with the results of binding assay (Fig. 4), these data show that BtR-175 is involved in PC pathogenicity.

## Discussion

Many Gram-positive and Gram-negative bacterial pathogens produce toxins that contribute to their virulence^31^. *Bb*, a typical natural pathogen of silkworms, is a model of insect host-pathogen interactions based on triggering a strong host response^20^. Here, we described the cloning and functional characterization of a novel toxin that contributes to the virulence of *Bb*, which we named PC. Bioinformatic analysis showed that PC does not exhibit any homology to other bacterial toxins and appears to be distinct from previously identified insecticidal PFTs such as Cry toxins (Fig. S3). Nevertheless, amino acid sequence analysis showed the presence of conserved residues in PC and *Bt* Cry toxins. Bioassays based on oral activity confirmed that this toxin isolated from *Bb* is an insecticidal toxin and that this toxin was lethal to *B. mori* (Figs 1 and 5).

How might PC induce silkworm death? The insect midgut represents the first line of resistance and triggers the immune response, which deploys multiple innate defence mechanisms to fight microbial intruders. However, many bacterial pathogens are equipped with clever infectious stratagems that circumvent these defence systems. The interplay between pathogens and their hosts involves the specific recognition of surface molecules that modulate cell recognition, membrane insertion, or internalization, which determine the fate of bacterial infections and disease outcomes. Toxins are important virulence factors for bacterial pathogenicity. Interactions with single receptors are a general strategy, although several toxins such as diphtheria, Cry, or aerolysin toxins target more than one surface molecule in their binding activities and action modes^8^. Here, we demonstrated that binding to BmAPN4 provokes the pathogenicity of PC (Figs 2 and 3), although the kinetic parameters of the interaction between PC with APN have not been fully analysed. APNs are known to play key roles in the mechanisms responsible for the toxicity of various classes of *Bt* Cry toxins^32^. Both the monomer and oligomer *Bt* Cry toxins interact with the APN receptor. The binding of oligomer to APN induces its insertion into cell membrane, leading to pore formation and cell lysis, which is a key determinant of toxicity^26^,^33^. APNs are highly abundant proteins that are anchored to membranes by a glycosyl phosphatidylinositol anchor^34^. In *Trichoplusia ni*, different APNs are associated with resistance to *Bt* Cry1Ac toxin^35^. In *Spodoptera exigua*, the absence of APN causes resistance to *Bt* Cry1Ca toxin^36^. In *B. mori*, BmAPN4 is specifically expressed in the midgu^28^,^37^, which explains why the injection of PC in the body cavity at high doses had no impact on silkworm survival (data not shown), while oral infection induced silkworm death. In this study, we report the discovery that BmAPN4, which is specifically expressed in the midgut, functions as a receptor and is involved in *Bb* recognition and toxin-related pathogenicity. Additionally, the binding of toxin to cell surface is the key step in causing toxicity. Thus, the loss or alteration of this receptor could play a pivotal role in the natural development of toxin resistance in silkworms.

The pathogenicity of *Bt* Cry toxin is the result of sequential interactions with at least two receptor molecules, cadherin and APN^26^. Interactions with APN concentrate *Bt* Cry toxin at the midgut brush border membrane, where the toxin can bind to cadherin receptor^33^. The binding of *Bt* Cry toxin to cadherin triggers toxin oligomerization to form a pre-pore structure, which interacts with APN and results in pore formation that leads to insect death^14^,^26^,^38^. Pores form in the silkworm intestinal epithelium after *Bb* oral infection, after which the infiltration becomes prominent, and material exchange throughout silkworm body is disrupted similar to *Bt* Cry toxin poisoning^20^. Nevertheless, whether the formation of pores related to APN occurs following the binding of PC to BmAPN4 is unknown. However, in this study we found that PC interacts with two regions of silkworm BtR-175 receptor that are located in the CR7 and CR12 cadherin repeats (Fig. 4), and does not bind to CR11. Cadherin repeats have been recognized as important regions of cadherin that mediate *Bt* Cry toxicity. In *Manduca sexta*, *Bt* Cry1A toxins bind to cadherin Bt-R_1_ receptor via interactions with CR7, CR11, and CR12 cadherin repeats, which are essential for toxicity^30^. Resistance to *Bt* Cry toxin in some insects is linked with mutations that disrupt a toxin-binding cadherin protein^39^. Using a CRISPR/Cas9 system to knock out the binding region of *BtR-175* gene for bioassay, we identified that BtR-175 mutant exhibited reduced PC pathogenicity *in vivo* (Fig. 5). The enhanced activity of PC in the presence of silkworm BtR-175 correlates with the mechanism of action of the toxin. These demonstrated that PC bound to silkworm BtR-175, which is located on the luminal membrane of midgut epithelial cells^40^, to induce host death.

Bacterial toxins secreted by pathogens diffuse towards the target cell by binding to specific receptors that are ultimately inserted in cell membranes and that form a pore^8^. Aerolysin, a pore-forming toxin, initially interacts with a high-abundance and low-affinity molecule and subsequently interacts with a low-abundance and high-affinity binding site^41^. A “ping pong” binding mechanism has been reported in which *Bt* Cry toxin is involved in the first binding event with the high-abundance and low-affinity APN receptor and then interacts with the high-affinity cadherin receptor^33^. APN is abundant in the *M. sexta* midgut, in contrast to cadherin, which is present at a much lower concentration^42^. Therefore, we speculate that PC binds to the high-abundance BmAPN4 receptor before interacting with Bt-R175. However, little is known regarding the structure of PC or its loop regions and receptor binding regions. In *Bt* Cry toxin, differences in sequence, length, and loop conformation are thought to be key determinants of its insect midgut BBMV binding ability and toxin selectivity. Thus, mapping the specificity of binding regions in PC will help to improve the applications of PC to insect pest control with *Bt* toxin.

**Figure 6:**
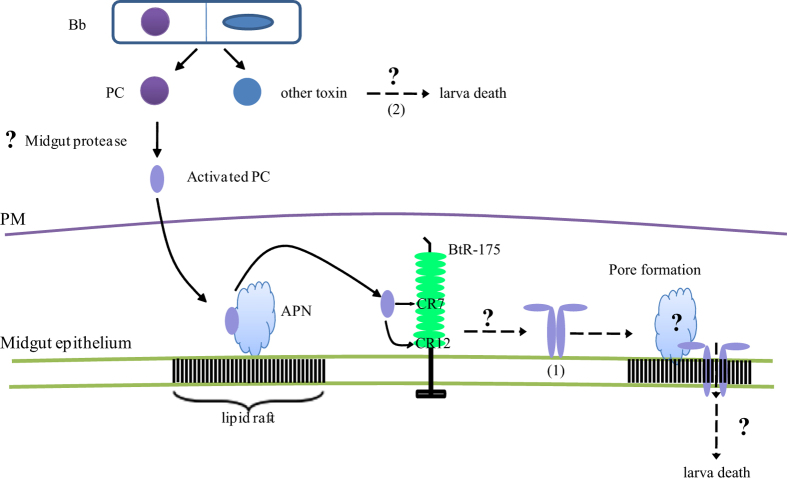
A schematic representation of the model for *B. bombysepticus* or PC causing damage to the silkworm midgut and translocating into the hemolymph. The larvae ingest *B. bombysepticus*, which produces parasporal crystal toxin (PC). PC can be digested by gut proteases. The digested PC pass through the peritrophic membrane (PM) to bind the high-abundance APN receptor, allowing the toxin to be located in close proximity to the membrane. This interaction is followed by high-affinity binding to the BtR-175 receptor by the cadherin fragments CR7 and CR12. Interactions with BtR-175 trigger the oligomerization of toxin that binds to the receptors and leads to pore formation (Pathway 1). However, unknown toxin(s) might also induce larvae death (Pathway 2). All of these toxins and receptors could work together, leading to aberrant gut infiltration and material exchange throughout the insect body, resulting in the death of larvae that is caused by *B. bombysepticus* infection.

The results shown here indicate that PC, a novel toxin, is pathogenic to silkworms. BmAPN4 primarily acts as a functional receptor that participates in PC pathogenicity and BtR-175 can interact with the toxin to affect pathogenicity. Based on our data, a schematic overview of *Bb* infection in the silkworm^20^ and previous studies of the mechanism of action of Cry toxins in lepidopterans at the molecular level^22^,^26^, we propose a schematic model for the process by which *Bb* damages the silkworm midgut and the haemolymph (Fig. 6). Larvae ingest *Bb*, which produce PC. PC can be digested by gut proteases. The digested PC passes through the PM to bind to the high-abundance APN receptor, after which the toxin is in close proximity to the membrane of the midgut. This interaction is followed by high-affinity binding to the BtR-175 receptor. Interactions with BtR-175 trigger oligomerization of the toxin, which then binds to the receptors and leads to pore formation (Pathway 1). This sequence of events is similar to the sequential model for Cry toxins activity proposed by Bravo *et al*.^22^,^26^,^43^. Additionally, unknown toxins might play a part in larval death (Pathway 2). In these models, all of the receptors would work together to disrupt the balance of infiltration and material exchange throughout the insect, resulting in larval death caused by *Bb* infection.

Remarkably, PC exhibits oral toxicity against *H. armigera* (Fig. 1) similar to *B. mori*. *H. armigera*, which is one of the most severe insect pests in Asia, attacks wheat, corn, vegetables, and cotton. The oral potency of PC and its insecticidal activity make it a good candidate for deployment against *H. armigera* and possibly other pest insect species. Because PC is a genetically encoded protein, engineering transgenes that encode this protein in plants should be possible. The most significant advancement in the use of bio-resources as bio-control agents in recent years is the use of transgenes encoding *Bt* Cry proteins, which have been incorporated into many crops to manage key insect pests successfully and to protect crops such as cotton and corn^14^. More than 420 million hectares are planted with *Bt* crops worldwide. Furthermore, in China, several *Bt*-producing crop varieties are waiting to be approved by the agricultural ministry for commercial use. However, to date, *Bt* Cry toxin resistance has been reported in some key pest species^15^,^44^,^45^, and the increasing use of *Bt* crops will accelerate the emergence of resistant insects and threaten long-term benefits. PC shows a pathogenic capacity towards three Cry1Ac-resistant *H. armigera* strains through oral infection (Fig. 1E), indicating that this toxin may provide farmers with a new weapon against the development of *Bt* Cry toxin resistance in insect pests. Thus, PC might be a good candidate for transgene engineering to replace or use in combination with *Bt* Cry toxins for integrated pest management systems with a strong biological control component.

The discovery of PC as a candidate for insecticides provides opportunities to develop PC into an effective insect pest control agent. Nevertheless, the most serious threat to the continued efficacy of PC is low insecticidal activity. PC has an LC_50_ value of 77 μg/per larvae. In contrast, Cry1A toxin has an LC_50_ value of 1 ng per square cm or a few hundred nanograms for *B. mori* instar larvae (less than 200 ng/cm^2^) indicating that PC is at least 400-fold less toxic^8^,^46^. The shortcoming of low toxicity of PC may be a limitation for its utility in pest control. Thus, one important future direction of research on PC is the provision of approaches to enhance its insecticidal efficacy. These possible strategies include the following. (i) The localization of the toxic portion of PC may be useful for enhancing PC activity. The goal of this strategy is to circumvent PC activation, resulting in improved toxicity against pests; thus, basic knowledge regarding the toxic portion of PC is helpful for the design of a toxin to control pests. (ii) Because the processing PC into its active form by insect proteases is crucial to its toxicity, enhanced processing by insect proteases may increase its efficacy against pests, such as the modification of PC to facilitate proteolytic activation. (iii) Binding to insect receptors is an important step in inducing toxicity; thus, increased binding to target insect receptors could improve insecticidal efficacy. Several approaches have been used to modify the binding affinity of *Bt* Cry toxins with the ultimate goal of producing designer toxins that target pests. According to these studies^14^,^47^, the alteration of PC binding affinity can be broken into the following categories: incorporation of binding fragments or domain swapping between PC and *Bt* Cry toxins, and site-directed mutagenesis of PC. This approach has provided important insight into PC residues that interact with insect midgut receptors. (iv) Phage display screens for mutated PC offer a potentially powerful method for engineering PC and for the subsequent selection of mutant PC with enhanced activity. Overall, these strategies for toxin modification will help to increase PC insecticidal efficacy against pests. Incorporation of these approaches into current management strategies will maximize the use of PC as a tool for crop protection and pest management.

Insect pests that lead to crop losses are a significant limiting factor in food production. In modern agriculture, insect pest control has undergone a revolution. In the United States, China, India, and Australia, the engineering of crop plants for enhanced resistance to insect pests has been a success for *Bt* crops that has resulted in reduced reliance on chemical insecticides. Second-generation *Bt* crops, termed pyramids, produce two or more *Bt* Cry toxins that are selected for resistance to one toxin that does not cause cross-resistance to the other toxins, which can delay or prevent the emergence of resistant insects^48^. In the past decade, farmers in Australia, India, and the United States have switched from planting first-generation transgenic crops to using pyramids against a particular pest^15^. Therefore, the goal of using PC transgenes in crops with *Bt* Cry toxins could be achieved by designing the toxin to enhance the efficacy of insect- and *Bt*-resistant pests, thus providing levels of crop protection that are more durable.

## Methods

### Insect and bacterial strains

Silkworm larvae (DaZao P50 strain) were reared on fresh mulberry leaves at a stable temperature of 25 °C. The *H. armigera* strains (LF, LF5, LF10, and LF240) were kindly provided by Prof. Kongming Wu (State Key Laboratory for Biology of Plant Diseases and Insect Pests, Chinese Academy of Agricultural Science, China) and reared using methods reported by Liang *et al*.^49^. The LF strain was started with approximately 100 3^rd^ to 6^th^ instars collected from *Bt* cotton in Langfang, Hebei Province, China in 1998. The LF5^50^, LF10 and LF240 strains were generated with resistance by selecting insects from LF strains grown with different concentrations of MVPII (Dow AgroSciences), a commercial formulation of Cry1Ac protoxin incorporated in the diet, over more than a decade. *Bb* was kindly provided by Prof. Yangwen Wang (Silkworm Disease Laboratory of Shandong Agriculture University, China).

### Purification of PC from *Bb*

The method for purifying PC was based on the procedure used for *Bt* Cry toxin purification^51^,^52^,^53^. *Bb* was grown on 100 μg/mL ampicillin at 37 °C. Cells were centrifuged at 8000 rpm for 20 min, and the pellet was washed three times with ice-cold 1 M NaCl and then washed five times with distilled water. The PC was solubilized in 50 mM sodium carbonate buffer (pH 9.5) containing 0.2% 2-mercaptoethanol and 25 mM EDTA on ice for12 h^54^. After the mixture was incubated, it was centrifuged at 8000 rpm for 30 min at 4 °C. The supernatant was subjected to a second round of centrifugation to remove insoluble components. The remaining supernatant was adjusted to pH 4.5 using 4 M CH_3_COONa-CH3COOH buffer and acetic acid, and was maintained on ice for 4 h. After the sample was centrifuged at 12,000 rpm for 30 min at 4 °C to remove the supernatant, the pellet was washed two times with distilled water. The PC protein was solubilized in 50 mM sodium carbonate buffer (pH 10) at 4 °C.

The solubilized PC protein was digested with trypsin at trypsin: toxin ratio of 1:25 (w/w) at 37 °C for 6 h. The proteins were purified by adding them to1/10 (v/v) 4 M CH_3_COONa-CH_3_COOH buffer and incubating them at 4 °C for 15 min before centrifugation at 12,000 rpm for 10 min. The pellet was then solubilized in distilled water. These steps were repeated five times. Finally, the pellet was solubilized in 50 mM sodium carbonate buffer. SDS–PAGE gels were run and then visualized using Coomassie Brilliant Blue (CBB) staining.

### Bioinformatics of PC

Signal peptide prediction was performed using the SignalP 4.1 Server (http://www.cbs.dtu.dk/services/SignalP/). The glycosylation sites were predicted by NetNGlyc 1.0 Server (http://www.cbs.dtu.dk/services/NetNGlyc/) and YinO Yang 1.2 (http://www.cbs.dtu.dk/services/YinOYang/). All sequences of *Bt* Cry toxin were downloaded from the databases (http://www.lifesci.sussex.ac.uk/home/Neil_Crickmore/Bt/index.html). A multipe sequence alignment and the neighbor-jioning (N-J) tree were constructed according to Lin *et al*.^28^.

### Expression vector construction and recombinant protein expression

The truncated ORF of *PC* and the cadherin repeats *CR7*, *CR11*, and *CR12* were cloned into pET-28a expression vectors with a 6×His tag on the N-terminal ends of the target sequences. BmAPN4 was cloned into a pGEX-4T-1 expression vector with a GST tag on the N-terminus. These recombinant plasmids were transformed into the *Escherichia coli* BL21 (DE3) strain to express PC, GST-tagged BmAPN4 (GST-BmAPN4), His-tagged CR7 (His-CR7), His-tagged CR11 (His-CR11), and His-tagged CR12 (His-CR12) proteins. The PC, His-CR7, His-CR11, and His-CR12 proteins were purified by affinity chromatography using Ni-NTA (GE Healthcare). GST-BmAPN4 was purified by GSTrapFF (GE Healthcare). All of these purified proteins were used for subsequent experiments.

### Construction of knock-out mutations

The plasmids of pUC57-hA4-Cas9 and pUC57-gRNA were kindly provided by Dr. Sanyuan Ma (State Key Laboratory of Silkworm Genome Biology, Southwest University, China). Guiding sequence of BtR-175 gRNA were synthesized and inserted to BbsI treated pUC57-gRNA, forming pUC-BtR-175. The microinjection of *B. mori* embryos, crossing strategy, T7 endonuclease I assay and sequencing of the mutations were performed according to the method of Ma *et al*.^55^.

### Determination of PC insecticidal activity on silkworm and *H. armigera* larvae

The mortality of silkworms after oral inoculation with PC was investigated. A fresh mulberry leaf was cut into square pieces 2 cm long and coated with a dose of 50 μg purified PC recombinant proteins per silkworm larva. Newly exuviated 3^rd^-instar and 5^th^-instar larvae that ate leaves with toxin were selected for continuous rearing. After 24 h, the larvae were transferred to a fresh diet (without toxin). Mortality was recorded after 4 days. We tested 30 larvae per test for each treatment, and this bioassay was replicated three times. The LC_50_ was calculated using GraphPad Prism software version 5.0 for Windows (GraphPad Software, San Diego, CA). Statistical analysis was performed with GraphPad using Student’s *t*-tests. No significant differences between the sessions were observed. *P* > 0.05 and statistically significant differences are indicated; ^*^ *P* < 0.05, ^**^P < 0.01.

For *H. armigera* bioassay, the method was based on the procedure by Cao *et al*.^25^. In brief, the 1^th^-instar susceptibility (LF) and three Cry1Ac-resistant larvae (LF5, LF10, and LF240) were placed individually on PC-contaminated artificial diet (doses of PC: 50 mg toxin/mL artificial diet) in plastic wells in 24-well plates. And 144 larvae were test per PC concentration. After five days, we recorded the mortality rate. The statistical analysis were performed according to above described.

### Cell transfection, immunofluorescence, and plasma membrane protein immunoblotting

Sf9 cells (Invitrogen) were cultivated in Grace medium (Gibco) supplemented with 10% foetal bovine serum, 0.35 g/L NaHCO_3_, and antibiotic-antimycotic (HyClone) at 27 °C. The ORF encoding the *BmAPN4* gene was cloned into the pSL1180[hr3-BmAct4-DsRed-SV40] plasmid between the *Bam*HI and *Not*I restriction sites, yielding the plasmid pSL1180[hr3-BmAct4-BmAPN4-SV40]. Cell transfection with the pSL1180[hr3-BmAct4-BmAPN4-SV40] plasmid was performed using Cellfectin II Reagent (Invitrogen) as described in a published protocol (Protocol Pub. No. MAN0007821). Immunofluorescence and immunoblotting of plasma membrane proteins were performed 48 h later. Cell membrane protein extraction from Sf9 cells was performed as described by Wang *et al*.^56^. Total membrane proteins were resolved by10% SDS-PAGE. After t the proteins were transferred to PVDF membranes, they were immunoblotted with anti-BmAPN4antibody (Invitrogen) or anti-Tubulin mAb (Sigma–Aldrich) following standard procedures. Immunofluorescence analysis was performed as described previously^57^ using FITC-conjugated goat anti-mouse IgG antibody.

### Far-western blot, co-immunoprecipitation, pull-down assay and ELISA binding assay

Far-western blots were performed according to the method of Wu *et al*.^58^. A summary of the Co-IP protocol is described below. The proteins (100 μg) were incubated with IP antibody for 1 h at room temperature. The complexes were bound to Protein A magnetic beads (Thermo Fisher Scientific) for 1 h at room temperature and then BS3 (Thermo Fisher Scientific) was used for cross-linking. After washing, the beads were incubated with PC (100 μg) overnight at 4 °C. The complexes were eluted after washing. The methods used for His pull-down experiments were the same as those described for the Pierce Protein Interaction Pull-Down Kit (Thermo Fisher Scientific). A summary of the ELISA protocol used was described previously by Pacheco^33^.

### Cytotoxicity assay of PC in Sf9 cells

Sf9 cells were harvested and transferred to a 48-well tissue culture dish (Costar) at 0.1 × 10^6^ cells per well. After the cells settled, they were transfected with pSL1180[hr3-BmAct4-BmAPN4-SV40]. After 48 h, the cell layer was overlaid with PC at 50 μg/mL in PBS after three gentle washes with PBS. The cells were incubated with the proteins for 6 h, after which they were observed under an Olympus TH4-200 microscope. The total number of cells per field was counted. Final values represent an average of 50 fields that were randomly selected for each treatment; each treatment was replicated three times. This assay measures the amount of stable cytosolic lactate dehydrogenase (LDH) released from the lysed cells using a coupled enzymatic assay that results in the conversion of a tetrazolium salt into a red formazan product according to the methods of Wang *et al*.^59^ and Tsuda *et al*.^60^.

### RNA extraction and quantitative real-time PCR after *Bb* challenge

RNA extraction and qRT-PCR analysis of the midgut expression pattern of the gene encoding BtR-175 after *Bb* infection were performed as described by Lin *et al*.^28^.

## Additional Information

**How to cite this article**: Lin, P. *et al*. PC, a Novel Oral Insecticidal Toxin from *Bacillus bombysepticus* Involved in Host Lethality via APN and BtR-175. *Sci. Rep*. **5**, 11101; doi: 10.1038/srep11101 (2015).

## Supplementary Material

Supplementary Information
