# Supplementary material for: PC, a Novel Oral Insecticidal Toxin from Bacillus bombysepticus Involved in Host Lethality via APN and BtR-175
Source: Sci Rep. 2015 Jun 9;5:11101. doi: 10.1038/srep11101 (PMC4460869; doi:10.1038/srep11101)
Supplement: Supplementary Information [file srep11101-s1.doc]

**Supplementary Information**

**PC, a Novel Orally Insecticidal Toxin from *Bacillus bombysepticus* Involved in Host Lethality via APN and BtR-175**

Ping Lin, Tingcai Cheng, Shengkai Jin, Yuqian Wu, Bohua Fu, Renwen Long, Ping Zhao, Qingyou Xia*

P.L. and T.C. contributed equally to this work.

**Table of Contents:**

**Supplementary Figure 1.** Phenotypes of PC-treated larvae.

(A) Larval phenotype after PBS-treated. (B) Death phenotypes after infection with PC at 24 h.

**Supplementary Figure 2.** Multi-sequence alignment of PC with *Bt* Cry1A-Cry28A.

Conserved sites are marked with shadow.

**Supplementary Figure 3.** Phylogenetic analysis of PC with *Bt* Cry1A-Cry28A.

Mega 4 with neighbour-joining bootstrapping (1000 interactions) was used to construct the tree.

**Supplementary Figures**


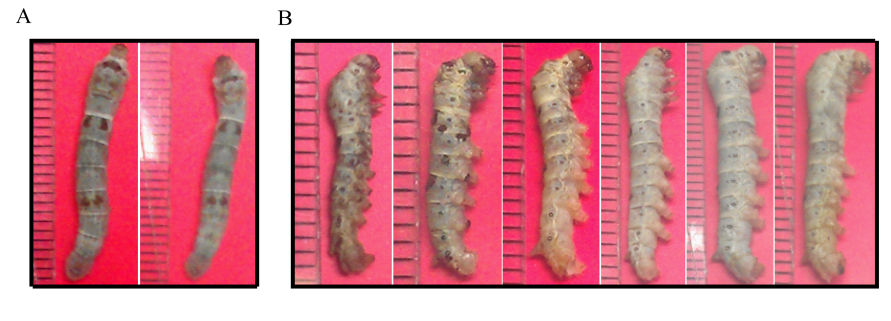


**Supplementary Figure 1.** Phenotypes of PC-treated larvae. (A) Larval phenotype after PBS-treated. (B) Death phenotypes after infection with PC at 24 h.


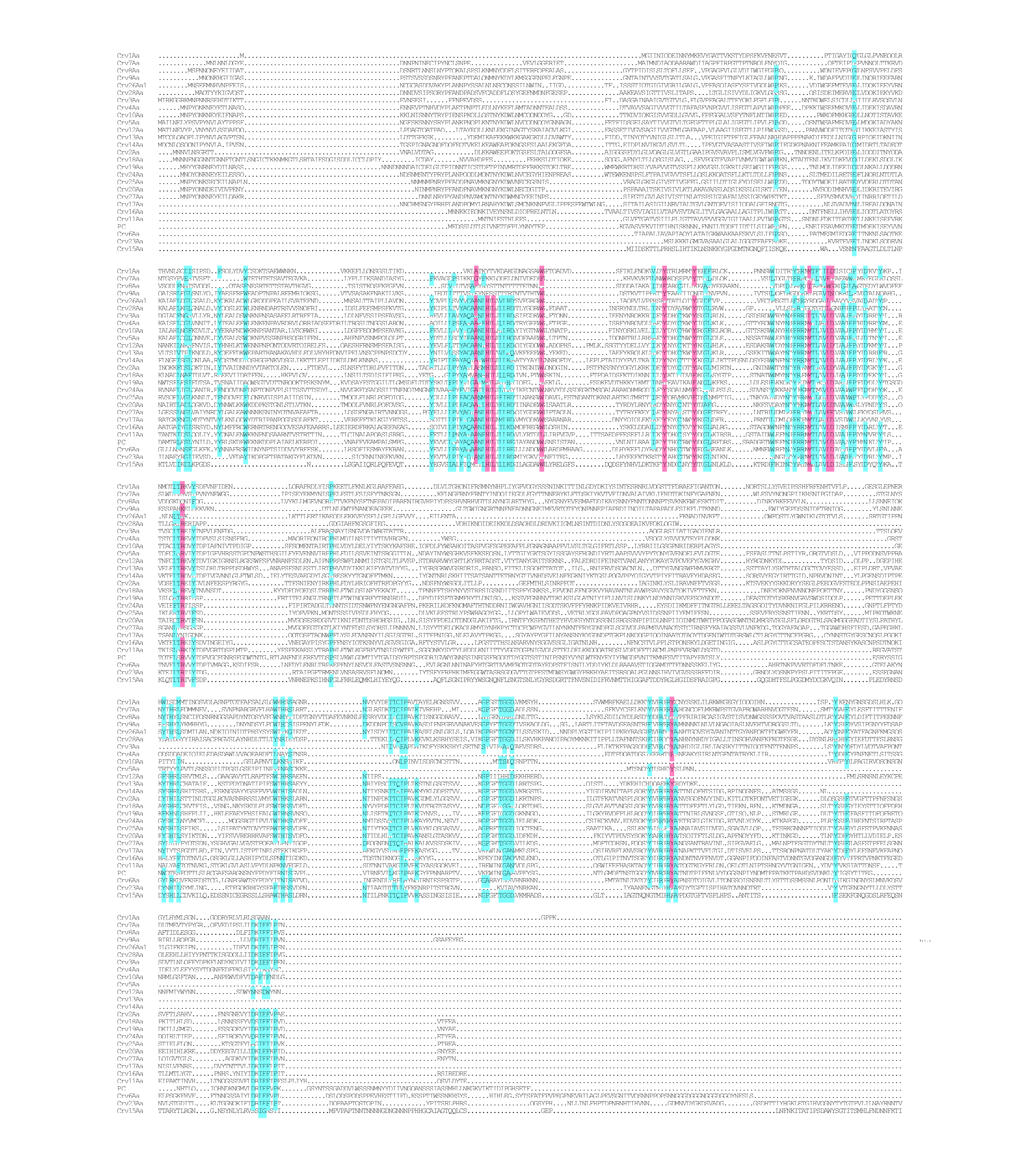


**Supplementary Figure 2.** Multi-sequence alignment of PC with *Bt* Cry1A-Cry28A. Conserved sites are marked with shadow.


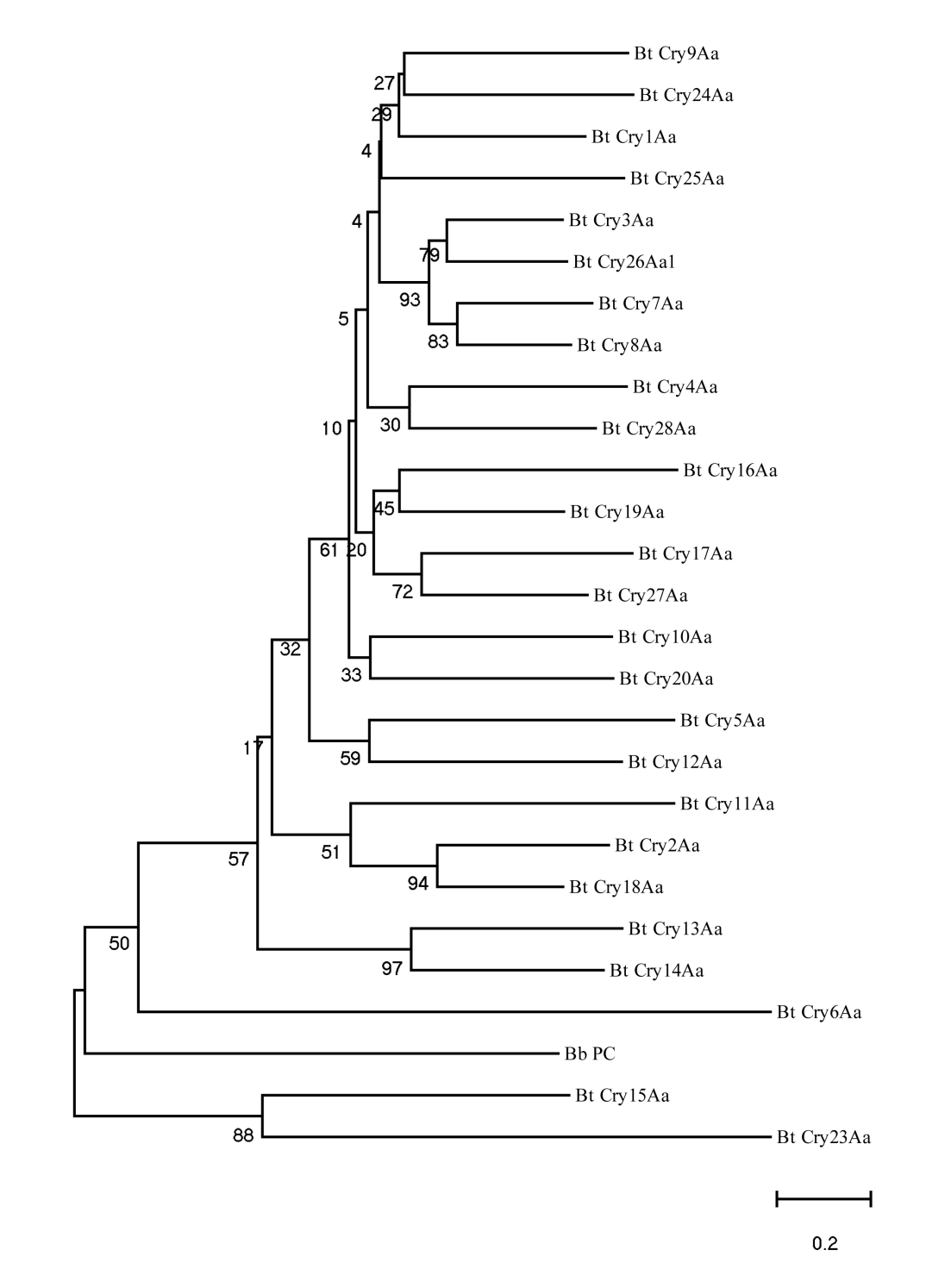


**Supplementary Figure 3.** Phylogenetic analysis of PC with *Bt* Cry1A-Cry28A.

Mega 4 with neighbour-joining bootstrapping (1000 interactions) was used to construct the tree.
